# Amino Acid–Fatty Acid Profile as a Novel Predictive Method in the Assessment of Diagnosis and Treatment Efficacy of Anxiety-Related Disorders and Mood Disorders

**DOI:** 10.3390/ijms27114705

**Published:** 2026-05-23

**Authors:** Mateusz Kowalczyk, David Aebisher, Jakub Szpara, Sara Czech, Edward Kowalczyk, Ireneusz Majsterek, Dorota Bartusik-Aebisher, Gabriela Henrykowska

**Affiliations:** 1 MediPsyche Medical Center, J. Kolinskiego 27, 91-849 Lodz, Poland; mateuszjerzykowalczyk@gmail.com; 2 Department of Photomedicine and Physical Chemistry, Collegium Medicum, Faculty of Medicine, Rzeszów University, 35-310 Rzeszów, Poland; 3 English Division Science Club, Collegium Medicum, Faculty of Medicine, Rzeszów University, 35-310 Rzeszów, Poland; js126214@stud.ur.edu.pl (J.S.); sc126240@stud.ur.edu.pl (S.C.); 4 Department of Pharmacology and Toxicology, Faculty of Medicine, Medical University of Lodz, T. Kosciuszki 4, 90-419 Lodz, Poland; edward.kowalczyk@umed.lodz.pl; 5 Department of Clinical Chemistry and Biochemistry, Faculty of Medicine, Medical University of Lodz, T. Kosciuszki 4, 90-419 Lodz, Poland; ireneusz.majsterek@umed.lodz.pl; 6 Department of Biochemistry and General Chemistry, Collegium Medicum, Faculty of Medicine, Rzeszów University, 35-310 Rzeszów, Poland; dbartusikaebisher@ur.edu.pl; 7 Department of Epidemiology and Public Health, Faculty of Medicine, Medical University of Lodz, T. Kosciuszki 4, 90-419 Lodz, Poland

**Keywords:** amino acid profile, fatty acid profile, fatty acid methyl esters, short-chain fatty acids, kynurenine pathway, tryptophan metabolism, metabolomics, lipidomics, major depressive disorder, generalized anxiety disorder, precision psychiatry, biomarkers, treatment response, gut–brain axis, multi-omics

## Abstract

**Major** depressive disorder (MDD) and anxiety disorders are increasingly understood as conditions involving complex metabolic dysregulation across multiple biological domains. This review aimed to synthesize current clinical and translational evidence on amino acid metabolism, lipid metabolism and short-chain fatty acids (SCFAs) as potential biomarkers, and components of integrative metabolic profiling in these disorders. A structured narrative approach was applied, focusing on studies assessing metabolomic alterations, their clinical correlates and their potential role in patient stratification, and treatment response. The available evidence indicates that amino acid disturbances, particularly within the tryptophan–kynurenine pathway, represent the most consistent and clinically interpretable findings. Lipid-related alterations, especially involving long-chain polyunsaturated fatty acids, provide complementary insights into membrane function, inflammation and neuroplasticity. In contrast, SCFAs appear to function as context-dependent markers rather than robust standalone biomarkers, with their clinical relevance depending on biological matrix, metabolic context and host–microbiota interactions. Importantly, most studies assess individual metabolites rather than integrated metabolic profiles, limiting their interpretability within a metabolomic framework. Overall, current evidence supports a shift toward integrative biomarker models that combine metabolic data with selected molecular and clinical parameters. Future research should focus on standardized, reproducible profiling approaches to enable biologically informed stratification and personalized treatment strategies.

## 1. Introduction

Recurrent major depressive disorder and depressive episode (MDD; ICD-10: F32–F33) as well as generalized anxiety disorder (GAD; ICD-10: F41.1) are among the most prevalent and most burdensome psychiatric entities on a global scale. According to the World Health Organization (WHO), depression affects approximately 4% of the global population, corresponding to more than 330 million individuals [1]. Anxiety disorders as a diagnostic category encompass approximately 360 million individuals worldwide, remaining the most common group of mental disorders [2]. These data clearly indicate that MDD and GAD constitute a population-level problem rather than rare clinical entities.

Major depressive disorder (MDD) and generalized anxiety disorder (GAD) remain among the leading contributors to the global burden of mental illness, with a substantial impact on long-term functioning and quality of life [3,4]. Despite this burden, both disorders remain biologically heterogeneous and current diagnostic frameworks still rely predominantly on symptom-based classification rather than objective biological stratification. Increasing evidence suggests that no single biomarker is likely to capture this complexity, and that integrative multi-omics approaches may provide a more clinically meaningful framework for precision psychiatry. In particular, converging alterations in amino acid and fatty acid metabolism and microbiota-derived signaling may reflect interconnected disturbances in neurotransmission, immune–metabolic regulation and treatment response. Therefore, the combined analysis of these pathways may help define biologically distinct patient subgroups and support the development of more robust predictive biomarkers for diagnosis and treatment stratification in mood and anxiety disorders [4,5,6,7].

From a clinical perspective, the high recurrence rate in MDD, the tendency toward chronicity in GAD, and the significant comorbidity between depressive and anxiety disorders are of key relevance. The co-occurrence of these conditions increases symptom severity, the risk of relapse, and reduces the likelihood of full remission. Importantly, despite the availability of numerous therapeutic options, a substantial proportion of patients fail to achieve a satisfactory clinical response. Treatment-resistant depression (TRD)—most commonly defined as a lack of response to at least two adequate trials of antidepressant pharmacotherapy—is estimated to affect ≥30% of patients with MDD [8]. This phenomenon is associated with an increased risk of hospitalization, suicide, and a marked rise in healthcare costs.

A key problem remains the biological and phenotypic heterogeneity of MDD and GAD. Current classification systems (ICD-10/ICD-11, Diagnostic and Statistical Manual of Mental Disorders, Fifth Edition—DSM-5) are based on symptom-based criteria that do not unequivocally reflect the molecular or neurobiological underpinnings of these disorders. Consequently, there are no routinely applied, validated biomarkers enabling stratification of biological disease subtypes, prediction of treatment response, or assessment of relapse risk. Current systematic reviews emphasize that despite intensive research on inflammatory, neurotrophic, metabolic, and genetic markers, no biomarker has yet been implemented into clinical standard practice in MDD [9,10].

As a consequence, the “trial-and-error” model in pharmacotherapy persists. Meta-analyses and umbrella reviews indicate that antidepressants significantly increase the likelihood of response and remission compared with placebo; however, this effect is characterized by substantial interindividual variability, and remission rates remain limited [11]. Similarly, in GAD, despite the confirmed efficacy of selective serotonin reuptake inhibitors (SSRIs) and serotonin–norepinephrine reuptake inhibitors (SNRIs), clinical response is heterogeneous, and a proportion of patients require multiple treatment modifications [12,13]. This variability suggests that the clinical phenotype may encompass biologically distinct disease subtypes that are not identified by current diagnostic criteria. Increasing evidence suggests that mood and anxiety disorders should be conceptualized as systemic disorders involving interdependent alterations in energy metabolism, neurotransmission pathways, the microbiota–gut–brain axis, and detoxification processes [14,15].

Within this framework, amino acid and fatty acid metabolism ceases to be merely a biochemical background and becomes a functional reflection of the neurobiological and immunometabolic state of the organism. Alterations in the availability of neurotransmitter precursors, the balance between pro- and anti-inflammatory metabolites, and the lipid signature may influence neuroplasticity, stress reactivity, and treatment response [16,17,18,19].

Simultaneously, microbiota-derived metabolites, including short-chain fatty acids (SCFAs), constitute a link between environmental factors and transcriptional and immunological regulation within the central nervous system [20,21].

Metabolomics, as a systemic analysis of low-molecular-weight metabolites, provides information on the current biochemical phenotype of the organism, integrating genetic, epigenetic, and environmental signals. The combination of metabolomic data—including the amino acid profile, SCFAs and fatty acid profiles—with the assessment of gene expression related to key metabolic and regulatory pathways establishes a framework for a multilayered approach that links the metabolic phenotype with molecular mechanisms underlying physiological and pathological processes. Such integration of metabolomics and PGx aligns with the concept of precision medicine (PM), the aim of which is the biological stratification of patients and the prediction of therapeutic response based on objective molecular parameters.

In light of accumulating evidence of systemic dysregulation of metabolic and regulatory axes in mood and anxiety disorders, integrated analysis of the amino acid–fatty acid profile and gene expression patterns may provide a basis for the development of molecularly grounded predictive models, the rationale and translational potential of which are further examined in the present review.

## 2. Amino Acid Profile (20 AA)

Amino acid profiling may provide clinically relevant information in mood and anxiety disorders because it reflects several biological domains at once, including neurotransmitter precursor availability, excitatory–inhibitory balance, immune activation and metabolic stress. Although a standard panel includes 20 proteinogenic amino acids, not all of them are equally informative from a neuropsychiatric perspective. In the context of MDD and GAD, the most biologically relevant alterations concern amino acids involved in glutamatergic and GABAergic transmission, monoamine synthesis, and inflammation-sensitive pathways of tryptophan and arginine metabolism. Among these, glutamate and γ-aminobutyric acid (GABA) are central to the regulation of excitation–inhibition balance. Glutamate is the principal excitatory neurotransmitter in the CNS and acts through ionotropic receptors, including N-methyl-D-aspartate receptors (NMDARs) and AMPA receptors, thereby influencing calcium-dependent signaling pathways involved in synaptic plasticity and neuronal adaptation [22]. By contrast, GABA, synthesized from glutamate by glutamate decarboxylase (GAD65/67), provides the main inhibitory counterbalance within cortical and limbic circuits. Reduced cortical GABA levels have been reported in MDD, particularly in prefrontal regions, supporting the view that altered excitation–inhibition balance is one of the neurochemical features of affective disorders [23,24].

Other amino acids are relevant not because they act directly as neurotransmitters, but because they determine the availability of key neuroactive compounds. Tryptophan and tyrosine are the principal precursors for serotonin and catecholamines, respectively. In particular, the transport of tryptophan across the blood–brain barrier depends on LAT1 (SLC7A5), which also carries branched-chain amino acids (BCAAs; leucine, isoleucine, and valine). This creates a competitive relationship in which peripheral amino acid composition may influence central tryptophan availability and, indirectly, serotonergic signaling [25,26].

A further pathway of interest involves arginine, which serves as the substrate for nitric oxide synthases (NOS1–3). Through its role in nitric oxide (NO) generation, arginine metabolism links amino acid availability with redox balance, neurovascular regulation, and synaptic signaling. Experimental and clinical data suggest that disturbances in the arginine–NO pathway, including altered regulation by asymmetric dimethylarginine (ADMA), may contribute to the pathophysiology of mood disorders [27].

Taken together, a broad amino acid panel should not be interpreted simply as a list of isolated metabolites. Its value lies in the possibility of capturing several interrelated pathophysiological domains at once. Within this framework, tryptophan metabolism appears to be of particular importance because it links immune activation with neurotransmitter balance and mitochondrial stress.

### 2.1. Pathophysiological Pathways: Switching of Tryptophan Metabolism

Under inflammatory conditions, interferon gamma (IFN-γ), interleukin-6 (IL-6), and tumor necrosis factor alpha (TNF-α) can induce indoleamine 2,3-dioxygenase 1 (IDO1) expression through JAK/STAT1- and NF-κB-dependent signaling [25,28]. IDO1 catalyzes the first and rate-limiting step of the kynurenine pathway (KP), shifting tryptophan metabolism away from serotonin synthesis and toward kynurenine production. As a result, substrate availability for serotonin synthesis may decrease, while the kynurenine/tryptophan (KYN/TRP) ratio increases. This ratio has been widely used as an indirect marker of inflammation-related tryptophan degradation in depression research [29].

Kynurenine crosses the blood–brain barrier via LAT1 and is then metabolized differently depending on cell type. In astrocytes, it is converted by kynurenine aminotransferases (KYAT1–4) into kynurenic acid (KYNA), a metabolite generally regarded as neuroprotective because it limits excessive glutamatergic signaling. In microglia, however, kynurenine is more readily directed by kynurenine 3-monooxygenase (KMO) toward 3-hydroxykynurenine and subsequently quinolinic acid (QA). QA is functionally important because it enhances NMDAR-mediated excitatory signaling, promotes oxidative stress, and may impair glutamate clearance, thereby contributing to excitotoxic conditions [29,30,31].

The relative balance between KYNA and QA is therefore often interpreted as an indicator of whether tryptophan metabolism is shifted toward a more neuroprotective or more neurotoxic/pro-inflammatory state. Under chronic inflammatory conditions, increased flux through the KMO-QA branch may contribute to glutamatergic dysregulation, mitochondrial burden, and altered stress-related neurobiology. At the same time, the kynurenine pathway also intersects with NAD^+^ biosynthesis, linking inflammation-related tryptophan metabolism with broader aspects of cellular energy regulation [32].

**Figure 1:**
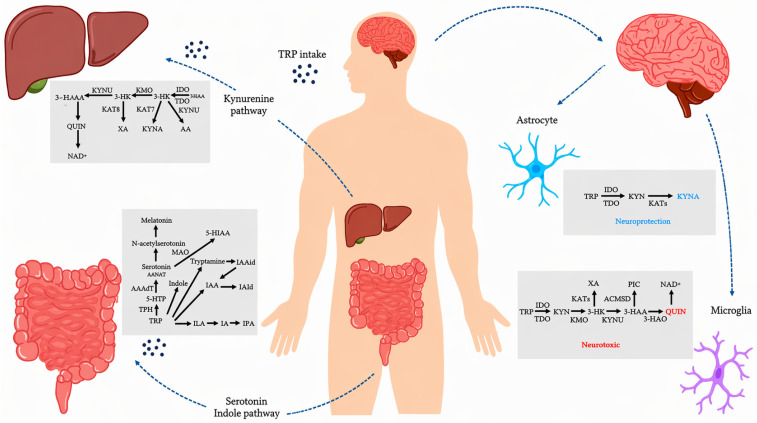
Overview of tryptophan metabolism along the kynurenine and serotonin/indole pathways within the gut–liver–brain axis, with potential relevance to anxiety-related and mood disorders. Dietary tryptophan (TRP) is metabolized through two major routes: (1) the kynurenine pathway, occurring in peripheral tissues and the central nervous system, and (2) the serotonin/indole pathway, involving the gastrointestinal tract, host metabolism and gut microbiota. In the liver, TRP is converted into kynurenine (KYN) via tryptophan 2,3-dioxygenase (TDO) and can be further metabolized into several downstream compounds, including kynurenic acid (KYNA) and quinolinic acid (QUIN). In the brain, glial cells contribute differentially to TRP metabolism; astrocytes preferentially produce KYNA, a metabolite considered to have neuroprotective properties, whereas microglia are more strongly associated with the formation of QUIN, a metabolite linked to neurotoxic and pro-inflammatory effects. In parallel, TRP may also be converted into serotonin (5-HT; 5-hydroxytryptamine) and further into melatonin, or transformed by gut microbiota into indole and its derivatives. These pathways illustrate the central role of TRP metabolism in the gut–brain axis, potentially linking intestinal, hepatic, immune and neural processes with the pathophysiology of anxiety disorders and mood disorders, as well as with treatment response [33]. **Abbreviations:** TRP, tryptophan; KYN, kynurenine; KYNA, kynurenic acid; QUIN, quinolinic acid; XA, xanthurenic acid; AA, anthranilic acid; 3-HK, 3-hydroxykynurenine; 3-HAA, 3-hydroxyanthranilic acid; NAD^+^, nicotinamide adenine dinucleotide; 5-HT, 5-hydroxyindole; 5-HIAA, 5-hydroxyindoleacetic acid; IAAld, indole-3-acetaldehyde; IAA, indole-3-acetic acid; IAld, indole-3-aldehyde; ILA, indole-3-lactic acid; IA, indoleacrylic acid; IPA, indole-3-propionic acid; KATs, kynurenine aminotransferases; KMO, kynurenine 3-monooxygenase; KYNU, kynureninase; IDO, indoleamine 2,3-dioxygenase; TDO, tryptophan 2,3-dioxygenase; TPH, tryptophan hydroxylase; MAO, monoamine oxidase.

For this reason, tryptophan metabolism may be viewed as one of the most informative amino acid-related pathways in mood and anxiety disorders. A summary of the principal enzymatic steps, cellular localization, and functional implications of this pathway is provided in Table 1. A schematic representation of tryptophan metabolism, including the kynurenine and serotonin–indole branches within the gut–liver–brain axis, is shown in Figure 1.

### 2.2. Clinical Evidence

One of the largest case–control studies, encompassing two independent cohorts of patients with MDD (total n = 511), demonstrated significant and replicable differences in the plasma amino acid profile between individuals in a current depressive episode and healthy controls [34]. Patients with MDD exhibited reduced concentrations of tryptophan, tyrosine, phenylalanine, methionine, asparagine, and histidine, accompanied by increased levels of glutamate and phosphoethanolamine. In integrated analysis, reduced methionine and elevated glutamate were strongly associated with an MDD diagnosis (*p* < 1.0 × 10^−6^), and the direction of changes was confirmed in both cohorts. This profile indicates concurrent dysregulation of the serotonergic, catecholaminergic, and glutamatergic axes, as well as potential disturbances in one-carbon metabolism and methylation processes. Additionally, leucine concentration correlated with the severity of anxiety symptoms, suggesting that amino acid metabolomics may also reflect the anxiety component in the course of depression.

The clinical relevance of amino acid profiling extends beyond case–control comparisons and may also be explored in relation to treatment response. In a randomized pharmacometabolomic study of patients with MDD treated with sertraline, early changes were observed in several circulating metabolites, including branched-chain amino acids (BCAAs), methionine, and tyrosine [35]. Lower BCAA levels were associated with better clinical outcome. However, such findings should be interpreted cautiously, as circulating amino acid concentrations are influenced by multiple non-specific factors, including nutritional status, energy balance, body composition, and systemic inflammation and therefore cannot be assumed to directly reflect central neurotransmitter function.

A similar caution applies to studies examining the relationship between tryptophan availability and antidepressant response. In one report, non-response was associated not simply with lower tryptophan concentrations, but with a less favorable balance between tryptophan and competing amino acids (CAAs) for LAT1-mediated transport across the blood–brain barrier, reflected in a lower TRP/CAA ratio [36]. This observation is of interest because it suggests that relative amino acid composition, rather than the absolute concentration of a single metabolite, may be more informative in some settings. At the same time, this type of peripheral marker should not be overinterpreted as a direct proxy of central serotonergic tone, since it may also be shaped by broader metabolic and inflammatory conditions.

More generally, longitudinal metabolomic studies suggest that some abnormalities in amino acid-related pathways may change during successful treatment, although the direction and specificity of these changes are not uniform across studies. In a serum metabolomics study of fluoxetine treatment, several metabolites distinguishing patients with depression from healthy controls showed partial normalization after 8 weeks of therapy, including markers linked to amino acid metabolism [37]. Likewise, in a more recent study comparing escitalopram, duloxetine, and cognitive behavioral therapy (CBT), changes in tryptophan-related metabolites were observed in the pharmacotherapy groups but not in the CBT group [38]. These findings do not indicate a single depression-specific biochemical signature, but rather support the view that different therapeutic interventions may engage partly distinct metabolic pathways, some of which may be detectable in peripheral biofluids.

Overall, the available evidence suggests that amino acid profiling may be more useful as a source of state-dependent or response-associated biomarkers than as a direct biochemical readout of specific neurotransmitter systems. Its translational value likely lies not in assigning individual metabolites to isolated neurochemical pathways, but in identifying broader patterns that may help to stratify patients or monitor biological change during treatment.

Particularly compelling evidence is provided by studies on treatment-resistant depression (TRD) and augmentation strategies. In a study involving ketamine administration, clinical response was associated with a rapid decrease in kynurenine concentration and increased arginine bioavailability as early as 4 h after infusion [39]. These findings indicate the involvement of the tryptophan–kynurenine and arginine–NO axes in the mechanism of rapid antidepressant action. In a study on electroconvulsive therapy (ECT), metabolites of the kynurenine pathway, including TRP, KYN, and the KYN/TRP and KYNA/KYN ratios, predicted the anti-anhedonic effect of ECT, and their changes correlated with symptom reduction [40]. Similarly, in a neuromodulation study in patients with bipolar depression, higher baseline quinolinic acid concentrations predicted improvement following active theta burst stimulation, but not in the placebo group [41]. These data suggest that kynurenine pathway metabolites may serve as stratification biomarkers for response to neuromodulatory interventions.

One of the strongest pieces of evidence supporting the predictive value of the amino acid profile is a secondary analysis of a randomized trial of N-acetylcysteine (NAC) augmentation in bipolar depression [42]. The baseline amino acid profile comprising nine metabolites explained 85% of the variance in improvement on the MADRS scale, and patients with lower baseline concentrations of free amino acids were more likely to respond to treatment. These findings indicate that systemic amino acid metabolism may serve as a stratification tool for therapies targeting the glutamatergic and redox systems.

The tryptophan–kynurenine axis also appears to be susceptible to immunometabolic interventions. Probiotic supplementation as an augmentation to SSRI treatment resulted in a significant reduction in kynurenine levels and improvement in cognitive function in patients with MDD [43], suggesting the possibility of modulating the kynurenine pathway via the gut–brain axis. In post-stroke depression, phototherapy was associated with increased tryptophan and BH4 concentrations, as well as improvement in the BH4/BH2 balance, accompanied by a reduction in inflammatory markers and clinical improvement [44]. Since BH4 is a key cofactor for tryptophan and tyrosine hydroxylases, these observations indicate a close relationship between amino acid metabolism, monoaminergic system activity, and inflammatory processes.

Collectively, the available clinical data indicate that mood disorders are characterized by a reproducible pattern of amino acid metabolism disturbances involving tryptophan, competing amino acids, methionine, and glutamate, and that relational metabolic indices (TRP/CAA, KYN/TRP, KYNA/KYN) demonstrate both diagnostic and predictive potential. The amino acid profile may stratify response to SSRIs, augmentation therapy, ketamine, ECT, and neuromodulation, and the kynurenine pathway emerges as a dynamic and intervention-sensitive component of depression pathophysiology. Notably, despite growing interest in immunometabolism in psychiatry, analogous metabolomic studies assessing the systemic amino acid profile in GAD are lacking, indicating a significant research gap and justifying the need for further studies integrating metabolomics with analysis of gene expression regulating metabolic pathways. A detailed summary of clinical studies and their potential biomarker relevance is presented in Table 2.

## 3. Short-Chain Fatty Acids (SCFAs) as Context-Dependent Gut–Brain Axis Markers

### 3.1. Biological Rationale

Short-chain fatty acids (SCFAs), primarily acetate, propionate and butyrate, are generated during microbial fermentation of indigestible carbohydrates in the intestine and represent one of the most frequently discussed classes of microbiota-related metabolites in gut–brain axis research. Beyond their role as a local energy source for colonocytes, SCFAs have been implicated in immune regulation, endothelial signaling, and epigenetic modulation, making them biologically relevant to neuroinflammatory and stress-related models of mood disorders [45].

Their effects are mediated mainly through two mechanisms: activation of G protein-coupled receptors (FFAR2/GPR43, FFAR3/GPR41, and GPR109A) and histone deacetylase (HDAC) inhibition, particularly in the case of butyrate and, to a lesser extent, propionate [46]. Through these pathways, SCFAs may influence transcriptional programs related to inflammatory signaling, oxidative stress, and cellular stress adaptation. Some reviews also suggest that HDAC-related effects of SCFAs may extend beyond histones and involve proteins relevant to tight junction stability and endothelial function [45,46,47].

Among the proposed CNS-relevant mechanisms, regulation of the microglial phenotype is one of the most frequently discussed. Experimental studies suggest that SCFA deficiency may be associated with altered microglial maturation and exaggerated inflammatory responsiveness, whereas SCFA exposure—particularly butyrate—has been linked to a less pro-inflammatory profile [46,47,48]. At the same time, these effects should not be treated as interchangeable with those of synthetic HDAC inhibitors, as available data indicate that butyrate may exert partly distinct molecular effects [49].

SCFAs have also been implicated in the regulation of blood–brain barrier (BBB) integrity. Experimental and review data suggest that microbiota-derived metabolites, including SCFAs, may influence the expression or localization of tight junction proteins such as claudin-5, occludin, and ZO-1, thereby potentially affecting endothelial barrier properties [47]. In cellular models, propionate has shown barrier-supportive effects, supporting the view that individual SCFAs may differ in their predominant biological actions [50]. More recent reviews have interpreted such findings as potentially relevant to persistent neuroinflammation and altered neurovascular signaling in affective disorders [51].

At the same time, SCFAs should not be interpreted as a uniform biochemical entity. Butyrate is most strongly linked to HDAC inhibition, propionate appears to show more consistent endothelial effects in vitro, whereas acetate, although quantitatively dominant in the circulation, appears to have more context-dependent systemic effects [46,50]. This distinction is relevant for biomarker interpretation, because aggregation of “total SCFA” values may obscure biologically different signals.

Importantly, the interpretation of SCFAs depends strongly on the biological matrix. Fecal SCFA concentrations more directly reflect colonic microbial fermentation, whereas circulating SCFA levels represent the net result of microbial production, intestinal absorption, portal transport, hepatic metabolism, and host intermediary metabolism [52,53,54]. This distinction is particularly relevant for acetate and formate, which may originate not only from microbial fermentation but also from host metabolic pathways [55,56].

Stable-isotope studies further demonstrate that the systemic availability of colon-derived SCFAs is both limited and metabolite-specific. In a controlled human study using colonic SCFA delivery, estimated systemic availability was approximately 36% for acetate, 9% for propionate and 2% for butyrate, reflecting substantial differences in intestinal absorption, hepatic extraction, and peripheral utilization [53]. These findings indicate that plasma SCFA concentrations do not directly reflect microbial production and should instead be interpreted as composite host–microbiota metabolic signals.

Microbial SCFA formation occurs through several distinct metabolic pathways. Acetate is produced primarily via acetyl-CoA and the Wood–Ljungdahl pathway, propionate via the succinate, acrylate, and propanediol pathways, and butyrate mainly from acetyl-CoA via butyryl-CoA [52,57]. These pathways differ across bacterial taxa and substrates, supporting the view that SCFA profiles represent pathway-dependent and context-specific metabolic outputs rather than uniform markers of microbial activity.

**Figure 2:**
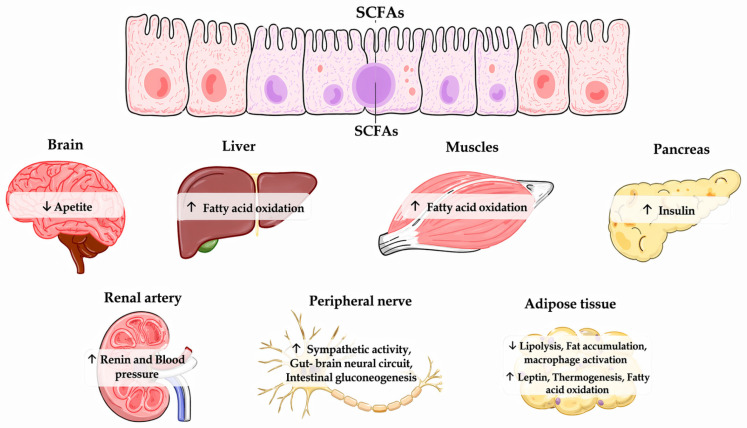
Schematic overview of the principal biological targets of short-chain fatty acids (SCFAs), with emphasis on gut–brain axis-relevant mechanisms. This figure should be interpreted as a conceptual overview of the principal biological targets of SCFA signaling rather than as evidence that acetate, propionate and butyrate exert identical effects or share identical transport routes. Individual SCFAs differ in microbial production pathways, receptor affinities, intestinal transport mechanisms, hepatic extraction, systemic availability and tissue-specific actions.

A schematic overview of these relationships is presented in Figure 2, which should be interpreted as illustrating the principal targets of SCFA signaling rather than a set of equally established effects in mood and anxiety disorders. In this context, the reference to brain function extends beyond appetite-related pathways and includes processes more directly relevant to the gut–brain axis, such as microglial reactivity, BBB maintenance, and stress-related neuroimmune signaling [47,48,49,50,51]. Likewise, the effects depicted in the liver and skeletal muscle should be understood as components of broader systemic immunometabolic regulation, rather than as isolated psychiatric mechanisms [45,46,47,50].

### 3.2. Clinical Relevance

Clinical studies examining SCFAs in mood and anxiety disorders remain heterogeneous, and their interpretation depends on clinical phenotype, biological matrix, and study design. As a result, SCFAs cannot currently be regarded as a well-established standalone “profile” analogous to amino acid or lipid panels. Rather, they may be better understood as context-dependent markers reflecting selected aspects of gut–immune–metabolic function. A major limitation for clinical translation is the lack of full methodological standardization in SCFA measurement. Comparability across studies is affected by differences in biological matrix, sampling conditions, dietary control, pre-analytical handling, extraction procedures, and analytical platforms. Accordingly, although SCFAs are biologically informative, their current reproducibility across laboratories remains insufficient to support their use as standardized standalone predictive biomarkers in routine psychiatric practice.

Importantly, from a metabolomic perspective, SCFAs should be interpreted as compositional patterns rather than isolated metabolites. Measures such as the relative proportions of acetate, propionate, and butyrate, as well as differences between fecal and circulating SCFAs, may provide more biologically meaningful information than absolute concentrations of single compounds. A major limitation of current studies is that most analyses focus on single SCFAs (most commonly butyrate), rather than integrated SCFA profiles.

Observational studies suggest that alterations in SCFA composition—particularly reduced butyrate and shifts in relative SCFA proportions—may co-occur with inflammatory subtypes of depression and with markers of intestinal barrier dysfunction [58]. Such findings are potentially relevant for endotype stratification, although they do not establish SCFAs as specific diagnostic biomarkers.

Interventional studies indicate that SCFAs may be more informative as response-associated markers than as universal therapeutic targets, particularly when interpreted as relative patterns rather than individual metabolites. For example, in an 8-week randomized trial of Limosilactobacillus reuteri as adjunctive treatment in MDD with low-grade inflammation, the probiotic did not show overall superiority over placebo for depressive outcomes, yet baseline levels and changes in selected organic acids were associated with symptom improvement in the active-treatment group [59]. This type of result suggests that SCFA-related measures may be more useful for identifying biologically responsive subgroups than for demonstrating uniform treatment effects across all patients.

More direct experimental evidence comes from studies in which SCFAs were administered to increase colonic and/or systemic exposure. In a triple-blind randomized trial, delivery of an SCFA mixture to the colon attenuated the cortisol response to psychosocial stress, suggesting a possible effect on HPA axis reactivity [60]. However, in a subsequent trial, colonic delivery of butyrate alone did not alter the acute stress response despite measurable biological effects on other domains, including fear-memory-related processes [61]. Together, these findings suggest that individual SCFAs may not be functionally interchangeable and that their relevance may depend on the specific biological or behavioral domain being assessed.

Additional pilot studies in populations with depressive and/or anxiety symptoms have reported concurrent shifts in microbiota composition together with changes in SCFA-related parameters [62]. However, these findings remain exploratory and are typically based on limited or indirect assessment of SCFA profiles, which restricts their interpretation in a metabolomic context.

Overall, current evidence supports the view that SCFAs are best interpreted not as a specific diagnostic signature of MDD or GAD, but as a context-sensitive translational component of the gut–brain axis. Their most plausible clinical utility may lie in biological stratification, particularly in inflammatory or metabolically burdened subgroups, and in monitoring biological response to interventions that modify microbiota-related or dietary pathways. At present, however, evidence remains insufficient to support routine use of SCFA measures as independent biomarkers in mood and anxiety disorders. Table 3 summarizes the principal findings of the studies discussed above, including the biological matrix used (feces/serum/plasma), direction of SCFA-related changes, and their possible biomarker interpretation. It should also be emphasized that evidence specifically addressing GAD as a diagnostic entity remains particularly limited, highlighting a clear need for future studies using standardized and parallel measurements of fecal and circulating SCFAs. Therefore, current clinical evidence on SCFAs is better interpreted as reflecting partial metabolic signatures rather than true SCFA “profiles” in the metabolomic sense.

SCFAs (acetate, propionate, and butyrate) are generated in the colon by microbial fermentation of indigestible carbohydrates and subsequently act on intestinal, immune, vascular, metabolic, and neural tissues. Their effects are mediated through receptor-dependent signaling (mainly FFAR2/GPR43, FFAR3/GPR41, and GPR109A) and receptor-independent epigenetic mechanisms, especially HDAC inhibition, which is most strongly associated with butyrate and, to a lesser extent, propionate. In relation to the gut–brain axis, the figure should be interpreted as illustrating both direct neurobiological effects (including modulation of microglial phenotype, BBB stability, and stress-/emotion-related brain function) and indirect peripheral pathways by which SCFAs shape the immunometabolic milieu relevant to affective symptoms. Thus, the effects shown for the brain, liver, muscle, adipose tissue, pancreas, renal/vascular compartment, and neural circuits should not be viewed as isolated outcomes, but rather as interconnected components of a broader microbiota-SCFA-immune-neurovascular signaling network [45,46,47,48,49,50,51,52,53,54,55,56,57].

## 4. Long-Chain Fatty Acid Profile (LCFA)

### 4.1. Membrane Lipids and Neuroplasticity (EPA, DHA, AA, and the Omega-6/Omega-3 Axis)

The fatty acid profile (particularly long-chain fatty acids, LCFA, including LC-PUFAs, most commonly in the phospholipid fraction, e.g., erythrocytes) should be considered a lipid membrane phenotype: on the one hand, it reflects long-term availability and incorporation of LC-PUFAs into phospholipids; on the other, it determines membrane biophysics and the availability of lipid precursors of signaling mediators that modulate neurotransmission, neuroinflammation, and neuroplasticity. From a molecular perspective, the three LC-PUFAs with the strongest mechanistic relevance are DHA (22:6n-3) as a structural component of synaptic membranes, EPA (20:5n-3) as a functional regulator of the inflammation-resolution axis, and AA (20:4n-6) as a key precursor of eicosanoids and lipid signaling molecules. DHA (relatively abundant in brain phospholipids), due to its six double bonds, strongly influences acyl chain packing, bilayer elasticity, and fluidity, thereby affecting membrane organization at the nanoscale, including the architecture of lipid microdomains (lipid rafts). Rafts serve as platforms for clustering receptors, ion channels, and adaptor proteins; their reorganization alters the probability of protein–protein and protein–lipid interactions, modulating signal transduction efficiency (GPCRs/kinases) and the stability of synaptic complexes. Reviews addressing the role of lipid microdomains in the brain emphasize that disturbances in membrane lipid composition translate into altered synaptogenesis and neuronal network formation, processes directly coupled to plasticity [63].

Additionally, membrane and cellular models have demonstrated that DHA significantly modifies raft properties by altering phase-ordering contrast, thereby influencing domain stability and membrane component diffusion [64]. Consequently, a low proportion of DHA in the fatty acid profile may be interpreted as an indicator of reduced “membrane competence” for adaptive synaptic reorganization, particularly within emotion-regulating circuits (PFC-hippocampus-amygdala), where rapid synaptic remodeling and long-term changes in synaptic strength are required.

EPA has a relatively lower representation in CNS phospholipids compared with DHA, yet it is critical as a “functional” factor shaping the immunometabolic environment that conditions plasticity. The mechanism involves competition of EPA with AA for COX/LOX enzymes, shifting the mediator profile toward a less pro-inflammatory state, and increasing the potential generation of specialized pro-resolving mediators (SPMs), thereby limiting persistence of inflammatory responses and secondary inhibition of plasticity. Clinically, it is relevant that the efficacy of EPA interventions may depend on inflammatory phenotype: in a “match-mismatch” study in patients with MDD on stable antidepressant treatment, add-on EPA was tested in the context of stratification by hs-CRP (low vs. high inflammatory status), supporting a model in which EPA acts preferentially in a subgroup with an inflammatory component [65]. At the level of fatty acid-based biomarkers, linking EPA to a “resolution phenotype” (less neuroinflammatory) is particularly useful, as it may indicate greater treatment responsiveness and reduced neuroinflammatory constraints on synaptic plasticity and treatment-induced neural adaptation.

AA remains a central node of the n-6 axis, as its release from phospholipids by phospholipase A2 initiates eicosanoid cascades (prostaglandins, leukotrienes, thromboxanes). Under conditions of chronic stress and low-grade inflammation (frequently described in depression and anxiety), excessive predominance of AA-derived pathways may promote persistence of inflammatory responses, microglial activation, and disruption of synaptic homeostasis, thereby creating an anti-plastic environment. At the same time, AA is not exclusively “negative”: it is also a precursor of lipid signaling molecules relevant for neuromodulation (including context-dependent eicosanoid pathways and interactions with lipids endogenously regulating excitability). Therefore, from a clinical perspective, the most accurate framework is not “AA per se,” but rather disproportion within the n-6/n-3 system and predominance of a pro-inflammatory phenotype [66,67].

In the context of neurotransmission—particularly the serotonergic axis—membrane lipids represent a regulatory layer for membrane proteins. Alterations in bilayer fluidity, thickness, and order, as well as raft reorganization, influence lateral diffusion kinetics, clustering, and conformational availability of receptors and transporters, potentially modifying signaling efficiency and responsiveness to monoamine-targeted treatments. In practice, a fatty acid profile phenotype characterized by lower DHA/EPA and relatively higher AA can be coherently integrated into a model in which increased pro-inflammatory mediators and oxidative stress impair synaptic function, neuroinflammation couples with tryptophan metabolism (via the kynurenine pathway) and substrate availability for serotonin synthesis, and structural-functional “rigidification” of membranes limits adaptive network plasticity in response to treatment [68].

For these reasons, the omega-6/omega-3 ratio (operationally: the relationship of total n-6, particularly AA, to EPA + DHA) serves as a mechanistically useful integrator of fatty acid profile findings. A higher n-6/n-3 ratio reflects a shift toward predominance of pro-inflammatory substrates and reduced availability of SPMs, potentially amplifying neuroinflammation, destabilizing synaptic homeostasis, and attenuating neuroplasticity. This model is supported by prospective data suggesting that a higher n-6/n-3 index may be associated with increased risk of mood disorders in high-risk populations [69]. Importantly, recent clinical studies have also linked modification of erythrocyte fatty acid profiles (following n-3 PUFA supplementation) with reduction in anxiety symptoms in the context of depression, directly supporting an approach in which the fatty acid profile represents not merely a “dietary background,” but a biomarker of a mechanistic axis (membrane–inflammation–neurotransmission–plasticity) [70].

### 4.2. Clinical Evidence

Within the domain of clinical evidence supporting the use of the fatty acid profile (including the omega-6/omega-3 axis) as a predictive component in mood disorders and comorbid anxiety, the collected studies converge on three consistent themes: the inflammatory phenotype as a condition for “unmasking” EPA effects, activation of inflammation-resolution pathways (SPMs) as a measurable mediating mechanism and potential response biomarker, and the “membrane” (erythrocyte) lipidomic component—together with related pathways (ECS, PLA2/COX-2, neurochemical markers)—as modulators of symptoms and therapeutic response.

The most biomarker-defined intervention is a randomized dose-finding trial in adults with MDD, overweight/obesity, and elevated inflammatory status (hs-CRP ≥ 3 mg/L), comparing 12-week EPA supplementation at 1 g/day, 2 g/day, and 4 g/day versus placebo, with prospectively defined endpoints including both clinical measures (IDS-C30) and functional indices of immune response (e.g., TNF in PBMCs and after stimulation). Despite the absence of consistent effects across all inflammatory markers (e.g., plasma IL-6), the 4 g/day arm demonstrated a moderate effect size versus placebo for clinical response (Cohen’s d ≈0.53). Crucially, at this dose, a significant association was observed between reduction in hs-CRP and decrease in depressive symptom severity. From a precision psychiatry perspective, this finding supports a model in which EPA may act preferentially in a subgroup with persistent low-grade inflammation, and inflammatory indices may serve as predictors or at least dynamic markers of response [71]. Interpretatively, this aligns with the hypothesis that shifting the lipidome toward n-3 dominance (and reducing functional predominance of the AA-dependent axis) does not necessarily translate into global cytokine changes in all individuals but may manifest as clinically meaningful effects in biologically “sensitive” subgroups.

Direct mechanistic corroboration of this observation is provided by two lipidomic analyses based on the same cohort, employing quantitative LC/MS to assess circulating EPA/DHA metabolites and specialized pro-resolving mediators. First, a clear dose- and time-dependent increase in EPA concentrations and its metabolites—particularly 18-HEPE (a precursor of E-series resolvins)—was demonstrated, along with enhanced conversion to RvE2–RvE3 and increased LXB4, accompanied by a reduction in plasma AA in the EPA arms [72]. This pattern confirms that 4 g/day EPA induces a biochemical phenotype consistent with a shift from inflammation “initiation/persistence” toward “resolution,” highly relevant for predictive biomarker concepts based on lipidomics rather than single cytokine measurements.

Second, in an analysis linking clinical response with mediators, responders in the 4 g/day arm exhibited significantly greater increases in 18-HEPE and 13-HDHA compared with non-responders, and the rise in 18-HEPE correlated both with hs-CRP reduction and IDS-C30 improvement [73]. From a scientific standpoint, this provides a valuable “bridge” between fatty acid metabolism and clinical outcome: not merely EPA exposure per se, but the organism’s capacity to activate the pro-resolving cascade (the “resolver” phenotype) may represent a mechanistically anchored predictor of therapeutic efficacy.

In somatically burdened populations, findings are more heterogeneous but continue to support a stratification-oriented approach. In a study of patients with cardiovascular disease (CVD) and comorbid MDD (12 weeks; 2 g EPA + 1 g DHA/day vs. placebo), no overall superiority was observed in reduction in total depression scores (HAMD/BDI); however, signals emerged in specific domains (e.g., improvement in the cognitive subscale at week 8), and after stratification, a greater reduction in core symptoms was noted in patients with very severe depression [74]. In a separate RCT in a similar CVD + MDD population focused on somatic symptoms and fatigue, n-3 PUFA supplementation was associated with earlier improvement in fatigue (week 4) and with biochemical shifts in lipid profile (increase in EPA, reduction in total omega-6). Subgroup analyses suggested associations between changes in EPA/n-3 PUFA, BDNF, and somatic symptom severity in younger patients [75]. Collectively, these data support the notion that “hard” depression endpoints may fail to reveal effects in multimorbid populations, whereas symptom domains (fatigue/somatization) and interactions between age and biomarkers may uncover biologically defined response subtypes.

Another consistent line of evidence concerns the endocannabinoid system (ECS) axis and regulation of gene expression relevant to phospholipid remodeling. In a study comparing three strategies (EPA, DHA, EPA + DHA; 12 weeks), higher cumulative remission rates were observed in EPA-containing arms compared with the DHA arm. Concurrently, a significant increase in plasma EPEA (eicosapentaenoyl ethanolamide) was noted in the EPA and EPA + DHA groups, with a positive association between EPEA levels and remission probability [76]. This finding provides clinical evidence that part of the effect of n-3 PUFAs may be mediated not only through eicosanoids/SPMs but also via lipid neuromodulators linked to the ECS, thereby directly connecting the lipidome with emotional regulation.

From a “lipid-gene axis” perspective, evidence that EPA and DHA differentially modulate gene expression is particularly relevant. In an RCT involving patients with acute depression, EPA (vs DHA) was associated with distinct modulation of cPLA2 expression, alongside parallel changes in erythrocyte PUFA composition and clinical improvement. This suggests that even when clinical improvement is directionally similar, the underlying molecular pathways may differ, with implications for selection of predictive biomarkers (e.g., indices of PLA2/COX pathway activity as switching nodes governing AA versus EPA/DHA availability) [77].

Directly addressing the thesis of the fatty acid profile as a predictive method is a study demonstrating that the anxiolytic effect of n-3 PUFAs in patients with first-episode, previously pharmacotherapy-naïve depression (co-treated with venlafaxine) is “mediated/manipulated” by changes in the erythrocyte fatty acid profile: supplementation increased the omega-3 index and EPA, reduced selected n-6 components, and improvement in anxiety symptoms correlated, inter alia, with decreases in selected saturated fatty acids and indices of desaturase activity (e.g., Δ5) [70]. This provides a direct argument that membrane parameters (RBC fatty acid profile) may function as biomarkers (monitoring and potentially predictive) for the anxiety domain in the course of MDD, rather than merely as markers of adherence or intake.

In pediatric populations, the most recent evidence is particularly important because a large, multicenter RCT (36 weeks; 1.5 g/day omega-3, EPA:DHA 2:1, as adjunctive therapy alongside standardized psychotherapy) showed no superiority over placebo in the trajectory of depression severity, response, or remission, despite a clear increase in the erythrocyte omega-3 index confirming adherence [78]. For the present review, this indicates that modification of the membrane lipidome (RBC) alone is not sufficient to achieve a clinical effect in a heterogeneous adolescent population with moderate-to-severe depression if the intervention is not biologically stratified (e.g., by inflammatory status or the capacity to generate resolution mediators). At the same time, the same research platform provided biomarker data indicating reduced anandamide (AEA) and hair cortisol concentrations in children and adolescents with MDD, as well as longitudinal associations between cortisol and symptom severity. This suggests that the HPA axis and ECS may represent more stable markers of disease phenotype and treatment monitoring than non-stratified n-3 supplementation “for all” [79].

Finally, independent of omega-3 intervention, a biomarker study in adolescents with first-episode, pharmacotherapy-naïve depression demonstrated significant differences in RvD1 and IL-4 concentrations versus controls and dynamic marker changes after fluoxetine treatment (decreases in RvD1, NLRP3, IL-1β, IL-18 and an increase in IL-4), supporting the concept of “dysregulated resolution” and the potential of pro-resolving mediators as diagnostic/monitoring markers in this age group [80].

Additionally, ^1^H-MRS studies in adolescent populations indicate that RBC EPA/DHA status and the AA/EPA ratio may be associated with markers of membrane and neuronal metabolism (e.g., choline, myo-inositol, Glx) in prefrontal/ACC regions, and supplementation may modulate some of these parameters despite an equivocal effect on the primary depression endpoint in placebo-controlled trials [81,82]. From a review perspective, this strengthens the hypothesis that the membrane lipidome is a coupling component linking immunometabolic signals with neurochemistry; however, translation into hard clinical outcomes requires appropriate population selection and stratifying biomarkers. Key methodological parameters, population characteristics, and the principal clinical and biomarker findings of the discussed studies are summarized in Table 4.

Although recurrent findings involving erythrocyte fatty acid composition and the omega-6/omega-3 balance appear biologically and clinically relevant, validated psychiatric cutoffs remain insufficiently established. At present, these measures are better interpreted as compositional and stratification-oriented signals rather than threshold-based standalone diagnostic tests. Their translational value likely lies more in relative pattern recognition than in universally accepted numerical cutoff values.

## 5. Integrative Biomarker Models in MDD

Recent integrative studies combining metabolomic, microbiome, and selected molecular markers in major depressive disorder (MDD) suggest that the biological architecture of depression is not adequately captured by isolated molecular variables, but rather by coordinated signatures involving lipid metabolism, amino acid metabolism, immune signaling, and microbiota-related pathways [83,84,85,86,87,88,89,90,91,92].

### 5.1. Amino Acids vs. Fatty Acids

In a longitudinal escitalopram-treated cohort, integration of shotgun metagenomics with plasma and fecal metabolomics demonstrated that antidepressant treatment was accompanied by a partial restoration of the metabolic profile. This was characterized by increases in amino acids that were reduced in MDD, including tryptophan, tyrosine, phenylalanine, alanine, and methionine, together with reductions in fatty acids that were elevated at baseline, such as palmitic, linoleic, oleic, and stearic acids [83].

Converging evidence from metabolome-wide and review-level studies further supports the central role of lipid and amino acid metabolism in depression. A large metabolome-wide investigation in UK Biobank reported that 191 of 249 metabolites were associated with lifetime MDD, with 129 associations persisting after adjustment for likely confounders. Mendelian randomization analyses suggested potentially causal effects of lower omega-3 fatty acids, lower docosahexaenoic acid, lower degree of unsaturation, and a higher omega-6:omega-3 ratio on MDD liability, whereas reverse causation from MDD to metabolite levels was not supported. Colocalization analyses further implicated the FADS locus, reinforcing the view that altered fatty acid metabolism may represent an upstream biological mechanism in depression rather than a purely downstream correlate [84].

In parallel, recent metabolomics syntheses have consistently highlighted amino acid and lipid-related pathways as the most reproducible biochemical domains in MDD, including tryptophan–kynurenine metabolism, glutamatergic and other amino acid pathways, glycerophospholipids, sphingolipids, fatty acids, phosphatidylcholines, and acylcarnitines. These findings support the rationale for integrative biomarker models that jointly capture amino acid and lipid disturbances rather than relying on single analytes [85].

### 5.2. Metabolites vs. Immune Markers

Integrative analyses have shown that clinically relevant heterogeneity within MDD can be resolved into biologically distinct subgroups with different outcome trajectories [86,87]. In a study combining metabolomics with cytokines and immune-cell phenotyping, three depression subtypes were identified. One subtype (MoS2) was characterized by markedly elevated triglycerides, increased regulatory T-cell abundance, and enrichment of nitric oxide metabolism, VEGF signaling, and ferroptosis pathways; this subtype showed the poorest antidepressant response and remained an independent predictor of nonresponse even after adjustment. In contrast, a second subtype displayed increased T-cell activity and higher levels of growth factors such as bFGF, HGF, and TGF-α and was associated with more favorable treatment outcomes, whereas a third subtype was characterized by elevated monocytes, reduced T-cell and Treg levels, and intermediate response [86]. In this context, cytokines should be interpreted as selected immune markers rather than proteomic data, because they represent targeted protein measurements rather than unbiased proteome-wide profiling.

Beyond subtype discovery, several studies indicate that treatment response is better represented by integrated biological profiles than by individual biomarkers [87,88]. A modeling study based on blood RNA sequencing, DNA methylation, and genotyping data identified response-associated “meta-phenotypes” derived from coordinated transcriptomic and epigenetic variation, with key pathways involving immune and inflammatory signaling (e.g., IL1RL1, IL5RA, PIK3R6, SMPD3) [88]. Similarly, in a multimodal longitudinal study, proteomics showed the strongest predictive performance among biological modalities, and the inclusion of proteomic features significantly improved prognostic accuracy beyond clinical variables. The most informative signals were related to inflammatory response and lipid metabolism, with fibrinogen emerging as the highest-ranking analyte [89].

### 5.3. Metabolites vs. Microbiome/Genetics

In the same longitudinal cohort, gut microbiome variation contributed to between-subject differences in circulating metabolites, particularly tryptophan-related compounds, and microbial functional features were associated with treatment remission, indicating joint host–microbiome contributions [83].

A related systems-level perspective emerged from a genomic–metabolomic network study, in which variants in circadian genes (*CLOCK*, *ARNTL*) were linked to phosphatidylcholines, kynurenine, amino acids, and carnitines. These metabolite–genotype relationships differed according to suicide attempt history, suggesting that metabolic signatures may vary across clinically meaningful MDD subphenotypes [87].

Studies incorporating the gut microbiome further support a coordinated microbiota–metabolism–inflammation axis in MDD, while also indicating that the metabolome may be more proximal to disease expression than taxonomy alone [90,91,92]. In a drug-naïve MDD cohort integrating metagenomics, plasma metabolomics, inflammatory cytokines, and structural MRI, MDD was associated with increased IL-1β, disturbances in lipid, vitamin, and carbohydrate metabolism, and altered gray matter volume, with microbial taxa showing extensive correlations with metabolites, inflammatory markers, neuroanatomical measures, and symptom severity [90].

In a more recent integrated microbiome–metabolomic study, plasma metabolites outperformed gut microbiota composition in distinguishing MDD from healthy controls, suggesting that the circulating metabolome may provide a more stable and clinically useful readout of gut-derived effects [91]. Consistent with this, another integrative study linked gut microbial variation to inflammatory markers, kynurenine metabolism, and cognitive performance, further supporting the involvement of the microbiota–immune–metabolic axis in depression [92].

Importantly, most available studies do not represent full multi-omics integration in the strict sense, but rather combine metabolomic data with selected molecular markers. Therefore, these approaches are better interpreted as integrative biomarker models rather than true multi-omics frameworks.

## 6. Conclusions

Major depressive disorder (MDD) and anxiety disorders are associated with complex and coordinated disturbances in multiple metabolic domains, most consistently involving amino acid metabolism, lipid metabolism, and selected components of the gut–brain axis. The available evidence suggests that these alterations are not adequately captured by single biomarkers but are better understood as integrated metabolic patterns reflecting underlying biological processes.

Among these domains, amino acid profiles—particularly those related to tryptophan–kynurenine metabolism—represent the most consistent and clinically interpretable signals. Lipid metabolism, especially long-chain polyunsaturated fatty acids (LC-PUFAs), provides complementary information related to membrane function, inflammation, and neuroplasticity. In contrast, short-chain fatty acids (SCFAs), while biologically relevant, currently lack sufficient standardization and consistency to be considered robust standalone biomarkers. Instead, they should be interpreted as context-dependent components of broader metabolic and microbiota-related processes, with their clinical relevance emerging primarily in specific biological subgroups.

Importantly, current evidence indicates that clinically meaningful information arises from the integration of metabolic data with selected molecular and clinical parameters rather than from single analytes. However, most available studies do not represent full multi-omics integration in the strict sense, but rather integrative biomarker approaches combining metabolomics with selected immune, genetic, or microbiome-related markers.

Overall, the most promising direction for future research lies in the development of standardized, reproducible metabolic profiling strategies that enable biological stratification of patients and support personalized treatment approaches. Achieving this will require harmonization of analytical methods, longitudinal study designs, and parallel assessment of multiple metabolic domains across clinically well-defined populations.

## Figures and Tables

**Table 1:** Enzymatic, cellular, and functional stages of TRP metabolism in the KP.

Stage	Enzyme (Gene)	Cellular Localization	Regulation	Product	Synaptic Effect	Mitochondrial Effect	Clinical Biomarker	Reference
TRP → N-formylkynurenine	IDO1 (IDO1)	Microglia, immune cells	IFN-γ, IL-6, TNF-α	KYN	↓ serotonin	-	↑ KYN/TRP	[25,28]
TRP → N-formylkynurenine	TDO2	Liver, CNS	Glucocorticoids	KYN	Stress modulation	-	KYN	[25]
KYN → KYNA	KYAT1–4	Astrocytes	Substrate-dependent	KYNA	NMDAR antagonist	↓ Ca^2+^ overload	KYNA	[31]
KYN → 3-HK	KMO	Microglia	Inflammation	3-HK	ROS	↑ oxidative stress	3-HK	[30]
3-HK → QA	KYNU, HAAO	Microglia	KMO-dependent	QA	NMDAR agonist	Mitochondrial dysfunction	QA	[29,30]
QA → NAD^+^	QPRT	Neurons	Metabolic	NAD^+^	-	Bioenergetic regulation	-	[32]

**Abbreviations:** TRP—tryptophan; KP—kynurenine pathway; IDO1—indoleamine 2,3-dioxygenase 1; TDO2—tryptophan 2,3-dioxygenase; KYN—kynurenine; KYAT1–4—kynurenine aminotransferases 1–4; KYNA—kynurenic acid; KMO—kynurenine 3-monooxygenase; 3-HK—3-hydroxykynurenine; KYNU—kynureninase; HAAO—3-hydroxyanthranilate 3,4-dioxygenase; QA—quinolinic acid; QPRT—quinolinate phosphoribosyltransferase; NAD^+^—nicotinamide adenine dinucleotide; IFN-γ—interferon gamma; IL-6—interleukin-6; TNF-α—tumor necrosis factor alpha; NMDAR—N-methyl-D-aspartate receptor; ROS—reactive oxygen species.

**Table 2:** Clinical studies assessing the plasma amino acid profile and kynurenine pathway metabolites as diagnostic, predictive, and treatment-response monitoring biomarkers in mood disorders.

Year	Population	Method	Key Clinical Findings	Biomarker Relevance	Reference
2018	229 MDD (dMDD + remission), 282 HC	HPLC (A), LC-MS (B)	↓ methionine, ↓ tryptophan, ↓ tyrosine, ↓ phenylalanine, ↑ glutamate; leucine correlated with psychic anxiety	↑ glutamate and ↓ methionine replicated in 2 cohorts; associated with MDD diagnosis (small–moderate effects)	[34]
2013	MDD, RCT, 4 weeks sertraline vs. placebo (longitudinal analysis; serum at weeks 0, 1, and 4)	GC-TOF-MS (serum metabolomics)	Changes in amino acids (BCAA, methionine, tyrosine); ↓ BCAA correlated with better response to sertraline; differences between drug and placebo response	BCAAs as potential predictive biomarkers of SSRI response; link between amino acid metabolomics and drug mechanism of action	[35]
2016	MDD (n = 50), 12 weeks standard treatment	Plasma TRP, CAA, TRP/CAA measurements + inflammatory markers	↑ CAA and ↓ TRP/CAA in non-responders; inverse correlation between CAA and IL-1RA	CAA and TRP/CAA ratio as potential biomarkers of non-response to treatment	[36]
2021	MDD (n = 20) vs. HC (n = 20); 8 weeks fluoxetine	UPLC-Q-TOF/MS (serum metabolomics)	16 biomarkers differentiating MDD vs. HC; partial normalization after treatment; disturbances in amino acid metabolism	Serum metabolomics as a tool for identification and monitoring of depression biomarkers	[37]
2025	MDD (n = 163), escitalopram vs. duloxetine vs. CBT; 12 weeks	Targeted LC–electrochemistry metabolomics (TRP/TYR pathways)	SSRI/SNRI: ↓ serotonin, ↑ TRP-derived indoles; distinct metabolites correlated with improvement depending on therapy	Treatment-specific metabolomic signatures; stratification potential	[38]
2018	TRD (n = 29) + healthy (n = 25); RCT, double-blind, crossover ketamine vs. placebo	Targeted plasma metabolomics (multipoint measurements)	Response to ketamine associated with ↓ kynurenine and ↑ arginine bioavailability (4 h post-infusion); no correlation between sphingomyelins and response	Tryptophan–kynurenine and arginine–NO pathway metabolites as potential biomarkers of early ketamine response	[39]
2025	TRD (n = 60), series of 8 ECT	Serum TRP, KYN, KYNA measurements + ratios	Higher TRP and lower KYNA in responders; TRP, KYN, and KYN/TRP predicted improvement in anhedonia	KYN pathway as a predictor of ECT response (anhedonia dimension)	[40]
2024	Bipolar depression (n = 37), cTBS vs. sham	ELISA (TRP, KYN, KYNA, QUIN)	Higher baseline QUIN predicted improvement in the active group	QUIN as a potential predictive biomarker of neuromodulation response	[41]
2021	Bipolar depression (n = 60); NAC vs. placebo; 16 weeks	GC-MS (untargeted metabolomics; 68 metabolites)	Lower baseline amino acids in responders; 9-amino-acid model explained 85% of MADRS improvement variance	Amino acid profile as a strong predictor of NAC response	[42]
2019	MDD (n = 60 analyzed); SSRI + probiotic vs. SSRI + placebo; 8 weeks	Plasma TRP–KYN metabolite measurements	↓ kynurenine (KYN) in probiotic group; ↑ 3HKYN:KYN ratio; improvement in cognitive function	Tryptophan–kynurenine pathway as a modifiable metabolic biomarker associated with clinical response	[43]
2024	Post-stroke depression (n = 100), phototherapy vs. standard treatment, 8 weeks	HPLC (Trp, BH4, BH2) + cytokines	↑ Trp and ↑ BH4; ↓ BH2; ↓ cytokines; improvement in HAMD/BDI	Trp and BH4/BH2 balance as potential markers for monitoring therapeutic response	[44]

**Abbreviations**: AA, amino acid; BCAAs, branched-chain amino acids; BDI, Beck Depression Inventory; BH2, dihydrobiopterin; BH4, tetrahydrobiopterin; CAAs, competing amino acids; CBT, cognitive behavioral therapy; cTBS, continuous theta burst stimulation; dMDD, current major depressive disorder; ECT, electroconvulsive therapy; ELISA, enzyme-linked immunosorbent assay; GC-MS, gas chromatography-mass spectrometry; GC-TOF-MS, gas chromatography-time-of-flight mass spectrometry; HAMD, Hamilton Depression Rating Scale; HCs, healthy controls; HPLC, high-performance liquid chromatography; IL-1RA, interleukin-1 receptor antagonist; KYN, kynurenine; KYNA, kynurenic acid; LC-MS, liquid chromatography-mass spectrometry; MADRS, Montgomery-Åsberg Depression Rating Scale; MDD, major depressive disorder; NAC, N-acetylcysteine; NO, nitric oxide; QUIN, quinolinic acid; RCT, randomized controlled trial; SNRI, serotonin–norepinephrine reuptake inhibitor; SSRI, selective serotonin reuptake inhibitor; TRD, treatment-resistant depression; TRP, tryptophan; TYR, tyrosine; UPLC-Q-TOF/MS, ultra-performance liquid chromatography coupled with quadrupole time-of-flight mass spectrometry; 3HKYN, 3-hydroxykynurenine.

**Table 3:** Summary of selected clinical studies assessing short-chain fatty acids (SCFAs) in relation to depressive and/or anxiety symptoms, with emphasis on clinically relevant populations and mechanistic models of SCFA-related biological pathways.

Year	Population	Method	Key Clinical Findings	Biomarker Relevance	Reference
2024	Patients with inflammatory depression	Microbiota analysis (stool), plasma SCFA, inflammatory and intestinal permeability markers	↑ *Bacteroides*, ↓ *Clostridium*; disturbed butyrate metabolism; correlation with inflammatory markers and intestinal permeability	SCFA (particularly the butyrate axis) as a marker of the inflammatory depression phenotype; potential role in stratification of the “inflammatory depression” subtype	[58]
2025	75 patients with MDD + overweight + hs-CRP ≥ 1 mg/L	8-week RCT, *L. reuteri* vs. placebo; fecal and plasma SCFA	No group-level effect on MADRS; ↓ MADRS correlated with ↑ fecal formic acid (*p* < 0.01) in probiotic group	Fecal formic acid as a potential response biomarker in inflammatory depression	[59]
2020	66 healthy men	Triple-blind RCT, 1 week; colon-delivered SCFA mixture (low and high dose)	↓ cortisol response to psychosocial stress; no mood changes	Direct evidence of HPA axis modulation by SCFA; correlation ↑ serum SCFA ↔ ↓ cortisol	[60]
2024	71 healthy men	Triple-blind RCT, 1 week; colon-delivered butyrate (5.28 g/day)	No effect on cortisol stress response; modulation of (subjective) fear-memory	↑ serum butyrate; no HPA effect → suggests SCFA mixture-specific effect	[61]
2025	79 individuals with mild-moderate depressive/anxiety symptoms	RCT, 8 weeks, BC99 vs. placebo; HAMD, HAMA; microbiota analysis; cytokines; neurotransmitters; SCFA	↓ HAMD (−2.40 points vs. placebo), ↓ HAMA (−5.53 points); higher response/remission (ns); ↑ SCFA production; ↑ SCFA-producing bacteria (*Faecalibacterium*, *Agathobacter*, *Dialister*, *Megamonas*)	SCFA as potential mediator of anti-inflammatory and neurotransmitter-related effects (IL-17 ↓, IL-10 ↑, GABA ↑)	[62]

**Abbreviations:** BC99, probiotic formulation BC99; GABA, gamma-aminobutyric acid; hs-CRP, high-sensitivity C-reactive protein; IL-10, interleukin-10; IL-17, interleukin-17; *L. reuteri*, *Lactobacillus reuteri*; MADRS, Montgomery–Åsberg Depression Rating Scale; MDD, major depressive disorder; RCT, randomized controlled trial; SCFA, short-chain fatty acids.

**Table 4:** Clinical studies evaluating omega-3 fatty acids (EPA/DHA) and lipidomic/fatty acid markers (including erythrocyte membrane indices and specialized pro-resolving mediators) as diagnostic biomarkers and predictors of treatment response.

Year	Population	Methodology	Key Clinical Outcomes	Biomarker Significance	Reference
2024	N = 72; first-episode, drug-naïve MDD + venlafaxine treatment	Randomized controlled trial (secondary analysis); erythrocyte FAME profiling; omega-3 Index; enzymatic activity analysis (Δ5-desaturase)	Improvement in anxiety symptoms correlated with ↓ C16:0, ↓ C18:0, and ↑ omega-3 Index	RBC fatty acid profile and Δ5-desaturase activity as potential predictors of anxiety symptomatology	[70]
2022	MDD with BMI > 25 kg/m^2^ and hs-CRP ≥ 3 mg/L; n = 61 (45 completers)	RCT; EPA 1–4 g/d; 12 weeks	4 g/d: 64% response vs. 40% placebo; correlation between reduction in hs-CRP and symptom improvement	hs-CRP as a potential predictor of response to omega-3	[71]
2021	N = 61 (42 in LC/MS analysis); MDD + BMI > 25 + hs-CRP ≥ 3 μg/mL	RCT; EPA 1–4 g/d; 12 weeks; LC/MS lipidomics	Dose-dependent ↑EPA, ↑ 18-HEPE, ↑ RvE2–3; ↓ AA; ↑ LXB4 (4 g/d)	18-HEPE and RvE2–3 as functional markers of inflammation resolution; shift in the omega-6/omega-3 axis	[72]
2023	N = 61 (42 in LC/MS analysis); MDD + BMI > 25; hs-CRP ≥ 3 μg/mL	RCT; EPA 1–4 g/d; LC/MS lipidomics (SPMs)	4 g/d: higher clinical response; increased 18-HEPE and 13-HDHA in responders	18-HEPE correlated with reductions in hs-CRP and symptom severity—candidate biomarker of response	[73]
2020	N = 59; CVD + MDD (61.5 ± 9 years)	RCT, 12 weeks; 2 g EPA + 1 g DHA; blood FA assessment	No overall effect; improvement in core symptoms in very severe MDD	Context: ↓ omega-3 and ↑ omega-6/omega-3 ratio in CVD + MDD; lack of robust predictive data	[74]
2023	N = 40; CVD + MDD (mean age 60 ± 9 years)	RCT, 3 g/d (2 g EPA + 1 g DHA), 12 weeks; assessment of PUFA, BDNF, and inflammation	Reduction in fatigue (Week 4); improvement in somatic symptoms in patients <55 years	↑ EPA and ↓ n-6 associated with symptom reduction; correlation of EPA and BDNF with clinical improvement	[75]
2019	N = 88 (85 completers); adults with MDD	RCT, 12 weeks; EPA 3 g/d vs. DHA 1.4 g/d vs. EPA + DHA; EC quantification (LC/MS)	Higher remission rates in the EPA groups; no superiority of DHA	↑ EPEA correlated with remission (HR 1.60); potential mediator and biomarker of response	[76]
2017	N = 27 MDD + 22 controls	12 weeks; EPA vs. DHA; FAME (RBC PUFA) + gene expression (cPLA2, COX-2, 5-HTT, TPH-2)	greater HAM-D reduction with EPA; ↑EPA in RBC	EPA ↑ cPLA2 expression (1.9×); molecular differences between EPA and DHA; integration of lipid profile and gene regulation	[77]
2026	N = 257; adolescents with moderate-to-severe MDD	RCT, 1.5 g/d EPA:DHA (2:1), 36 weeks; omega-3 Index (RBC)	No differences vs. placebo in CDRS-R, response, or remission	Increase in omega-3 Index without clinical effect; underscores the need for a biomarker-guided approach	[78]
2025	N = 110 MDD (8–17 years) + 127 controls	Hair AEA and cortisol; cross-sectional and longitudinal analysis	↓ AEA and ↓ cortisol in MDD; negative association between cortisol and symptom severity	AEA and cortisol as diagnostic and monitoring biomarkers in pediatric MDD	[79]
2024	N = 48 MDD (first episode, drug-naïve) + 30 HC; adolescents	Measurement of RvD1, NLRP3, IL-1β, IL-18, IL-4; before and after fluoxetine	RvD1 ↑ in MDD; correlation with HDRS; decrease in RvD1 and NLRP3 after treatment	RvD1 as a potential diagnostic biomarker and indicator of treatment response	[80]
2020	N = 56 (42 completers); adolescents with MDD, high familial risk for BD-I	RCT, 12 weeks; 2100 mg/d; RBC PUFA + ^1^H-MRS	No differences in CDRS-R; improvement in CGI	RBC n-3 PUFA ↑; ACC Cho correlated with symptom severity and PUFA changes	[81]
2016	N = 14; adolescents with MDD	10 weeks; FO 2.4 vs. 16.2 g/d; RBC FA + ^1^H-MRS (ACC, DLPFC)	Significant symptom reduction in the high-dose group	↑ EPA/DHA and ↓ AA in RBC; AA/EPA correlated with Cho in DLPFC; association between lipid profile and glutamatergic metabolites	[82]

**Abbreviations:** AA, arachidonic acid; ACC, anterior cingulate cortex; AEA, anandamide; BD-I, bipolar disorder type I; BDNF, brain-derived neurotrophic factor; BMI, body mass index; CDRS-R, Children’s Depression Rating Scale–Revised; CGI, Clinical Global Impression; COX-2, cyclooxygenase-2; cPLA2, cytosolic phospholipase A2; CVD, cardiovascular disease; DHA, docosahexaenoic acid; DLPFC, dorsolateral prefrontal cortex; EC, endocannabinoid; EPA, eicosapentaenoic acid; EPEA, eicosapentaenoyl ethanolamide; FA, fatty acid; FAME, fatty acid methyl ester; FO, fish oil; HAM-D/HAMD, Hamilton Depression Rating Scale; HC, healthy controls; HDRS, Hamilton Depression Rating Scale; hs-CRP, high-sensitivity C-reactive protein; IL-1β, interleukin-1 beta; IL-4, interleukin-4; IL-18, interleukin-18; LC/MS, liquid chromatography–mass spectrometry; LXB4, lipoxin B4; MDD, major depressive disorder; NLRP3, NOD-like receptor family pyrin domain containing 3 inflammasome; PUFA, polyunsaturated fatty acid; RBC, red blood cell; RCT, randomized controlled trial; RvD1, resolvin D1; RvE2–3, resolvin E2/E3-related metabolites; SPMs, specialized pro-resolving mediators; TPH-2, tryptophan hydroxylase 2; 5-HTT, serotonin transporter; 18-HEPE, 18-hydroxyeicosapentaenoic acid; 13-HDHA, 13-hydroxydocosahexaenoic acid; ^1^H-MRS, proton magnetic resonance spectroscopy; Δ5-desaturase, delta-5 desaturase; ↑ increased; ↓ decreased.

## Data Availability

No new data were created or analyzed in this study.
